# Synthesis and structure of two isomers of a molybdenum(II) 2-butyne com­plex stabilized by bioinspired *S*,*N*-bidentate ligands

**DOI:** 10.1107/S2053229622002029

**Published:** 2022-03-05

**Authors:** Madeleine A. Ehweiner, Ferdinand Belaj, Nadia C. Mösch-Zanetti

**Affiliations:** a Institute of Chemistry, Inorganic Chemistry, University of Graz, Schubertstrasse 1, 8010 Graz, Austria

**Keywords:** isomer, 2-butyne com­plex, molybdenum(II), benzene­thiol, crystal structure

## Abstract

The synthesis and structural determination of two isomers of the molybdenum(II) com­plex (η^2^-but-2-yne)carbonyl­bis­[2-(4,4-dimethyl-4,5-di­hydro-1,3-oxa­zol-2-yl)benzene­thiol­ato-κ^2^ *N*,*S*]molybdenum(II), [Mo(C_11_H_12_NOS)_2_(C_4_H_6_)(CO)] or Mo(CO)(C_2_Me_2_)(S-Phoz)_2_, are presented. The *N*,*N*-*cis*–*S*,*S*-*trans* isomer **1** shows quite different bond lengths to the metal atom [Mo—N = 2.4715 (10) *versus* 2.3404 (11) Å; Mo—S = 2.4673 (3) *versus* 2.3665 (3) Å]. In the *N*,*N*-*trans*–*S*,*S*-*cis* isomer **2**, which is isotypic with the corresponding W com­plex, the Mo—N bond lengths [2.236 (2) and 2.203 (2) Å], as well as the Mo—S bond lengths [2.5254 (8) and 2.5297 (8) Å], are almost the same.

## Abstract 2

Two isomers of the molybdenum(II) com­plex Mo(CO)(C_2_Me_2_)(S-Phoz)_2_ [S-Phoz is 2-(4,4-di­methyl­oxazolin-2-yl)thio­pheno­late] have been synthesized and characterized by X-ray diffraction at 100 K and by spectroscopy (NMR and IR). They show quite different Mo—N and Mo—S distances.

## Introduction

In order to explore the inter­action of Mo and W centres with acetyl­ene (C_2_H_2_), which is accepted as a substrate by the tungstoenzyme acetyl­ene hydratase (Schink, 1985 ▸; Rosner & Schink, 1995 ▸), our group has focused on the synthesis of W^II^ and Mo^II^ com­plexes containing bioinspired *S*,*N*-bidentate ligands and their subsequent oxidation to the respective W^IV^ and Mo^IV^ com­plexes. Although *N*-donor ligands are not the closest structural mimics of the di­thiol­ene ligands in the active site of acetyl­ene hydratase (Seiffert *et al.*, 2007 ▸) and other members of the dimethyl sulfoxide (DMSO) reductase enzyme family (Seelmann *et al.*, 2020 ▸), the use of these ligands has resulted in the discovery of new reactivities at W centres (Vidovič *et al.*, 2019 ▸; Ehweiner *et al.*, 2021*c* ▸), the isolation of a so-far-elusive Mo^IV^ C_2_H_2_ com­plex (Ehweiner *et al.*, 2021*a* ▸) and a detailed com­parison of W and Mo com­plexes with a variety of coordinated alkynes (Ehweiner *et al.*, 2021*b* ▸). One of the early publications of our group in this research field focused on the reversible activation of C_2_H_2_ at a W^IV^ centre coordin­ated by two 2-(4,4-di­methyl­oxazolin-2-yl)thio­pheno­late (S-Phoz) ligands (Peschel *et al.*, 2015*a* ▸). Thereafter, the re­ver­sible binding of C_2_Me_2_ and C_2_Ph_2_ (Peschel *et al.*, 2019 ▸) was investigated, with a particular focus on the flexibility of the S-Phoz ligand. The latter has also found application in Ni, Pd and Pt com­pounds (Peschel *et al.*, 2015*b* ▸; Holzer *et al.*, 2018 ▸), as well as in Zn (Mugesh *et al.*, 1999 ▸) and Fe (Bottini *et al.*, 2010 ▸) com­plexes.

Herein we report an improved synthetic procedure for Mo(CO)_2_(S-Phoz)_2_ and the preparation and structural characterization of carbon­yl(η^2^-1,2-di­methyl­ethyne)[2-(4,4-di­methyl­oxazolin-2-yl)benzene­thiol­ato-κ^2^ *N*,*S*]molydbenum(II), Mo(CO)(C_2_Me_2_)(S-Phoz)_2_, which forms two isomers (**1** and **2**) in solution, as well as in the solid state (see Scheme 1). This behaviour is different from that observed for the W variant which crystallized solely as the *N*,*N*-*trans* isomer and showed the presence of a second isomer in solution only to a minor extent. 
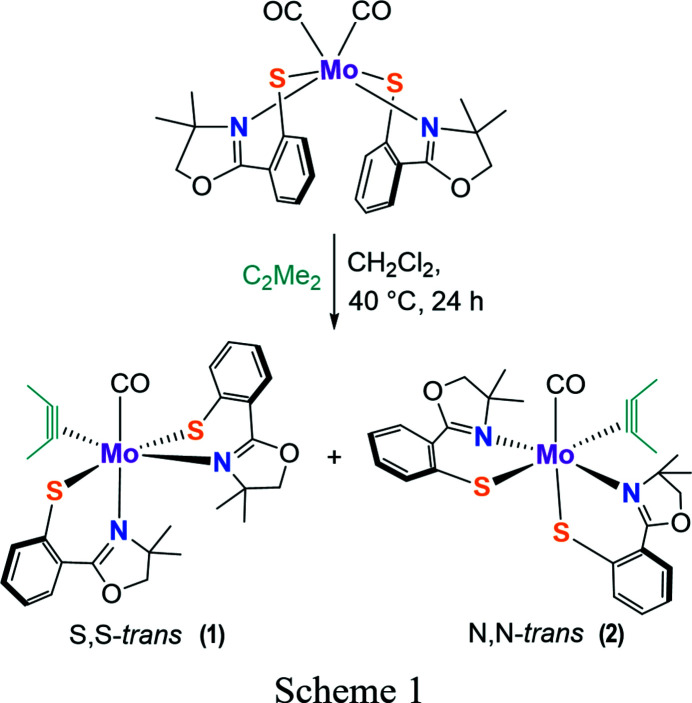


## Experimental

Synthetic manipulations were performed under a nitro­gen atmosphere using standard Schlenk and glove-box techniques. Solvents were purified *via* a Pure Solv Solvent Purification System. Chemicals were purchased from commercial sources and used without further purification. The precursor MoI_2_(CO)_3_(NCMe)_2_ was synthesized according to a literature procedure (Baker *et al.*, 1986 ▸). For the synthesis of Mo(CO)_2_(S-Phoz)_2_, a slight modification of a published procedure was used (Peschel *et al.*, 2013 ▸). ^1^H NMR spectra were recorded on a Bruker Avance III 300 MHz spectrometer at ambient tem­per­ature and are referenced to residual protons in the solvent. The multiplicity of peaks is denoted as singlet (*s*), doublet (*d*), doublet of doublets (*dd*) or multiplet (*m*). NMR solvents were stored over mol­ecular sieves. Solid-state IR spectra were measured on a Bruker ALPHA ATR–FT–IR spectrometer at a resolution of 2 cm^−1^. The relative intensity of signals is declared as strong (*s*), medium (*m*) and weak (*w*). Electron impact mass spectroscopy (EI–MS) measurements were performed with an Agilent 5973 MSD mass spectrometer with a push rod.

### Synthesis and crystallization

#### Preparation of Mo(CO)_2_(S-Phoz)_2_

A solution of Li(S-Phoz) (853 mg, 4.00 mmol) in MeCN (8 ml) was added dropwise to a solution of MoI_2_(CO)_3_(NCMe)_2_ (1.03 g, 2.00 mmol) in MeCN (8 ml). The resulting blood-red solution was stirred for 2 h at 35 °C, whereupon the solvent was removed by evaporation. The residue was suspended in toluene (20 ml) and the resulting suspension was filtered through Celite. The blood-red filtrate was then evaporated to dryness. After repeated recrystallization from CH_2_Cl_2_/heptane at −25 °C, Mo(CO)_2_(S-Phoz)_2_ (yield 790 mg, 70%) was ob­tained as dark red crystals. NMR and IR data are in agreement with previously published results (Peschel *et al.*, 2013 ▸).

#### Preparation of Mo(CO)(C_2_Me_2_)(S-Phoz)_2_

Mo(CO)_2_(S-Phoz)_2_ (339 mg, 0.60 mmol) was dissolved in CH_2_Cl_2_ (20 ml), whereupon 2-butyne (0.38 ml, 4.80 mmol) was added to the solution at 0 °C under stirring. The cooling bath was removed and the solution was heated under reflux for 24 h. Evaporation of the solvent gave a dark brown powder. Single crystals suitable for X-ray diffraction were obtained from CH_2_Cl_2_/heptane solutions at −35 °C. Crystals of both isomers (green plates of **1** and yellow needles of **2**) were obtained from the same batch. The product is very sensitive to air and should be stored in a glove-box.

#### Analytical data

^1^H NMR for **1** (CD_2_Cl_2_, 300 MHz, *S,S-trans* isomer, 34%): δ 8.07 (*dd*, *J* = 8.1, 1.1 Hz, 1H, PhH), 7.78–7.72 (*m*, 3H, PhH), 7.35 (*dd*, *J* = 7.8, 1.1 Hz, 1H, PhH), 7.32–7.27 (*m*, 2H, PhH), 7.21–7.01 (*m*, 1H, PhH), 4.46 (*d*, *J* = 8.2 Hz, 1H, CH_2_), 4.18 (*d*, *J* = 8.1 Hz, 1H, CH_2_), 4.11 (*d*, *J* = 8.3 Hz, 1H, CH_2_), 3.78 (*d*, *J* = 8.2 Hz, 1H, CH_2_), 2.70 (*s*, 3H, C≡CCH_3_), 2.55 (*s*, 3H, C≡CCH_3_), 1.89 (*s*, 3H, CH_3_), 1.81 (*s*, 3H, CH_3_), 1.57 (*s*, 3H, CH_3_), 1.44 (*s*, 3H, CH_3_); ^1^H NMR for **2** (CD_2_Cl_2_, 300 MHz, *N*,*N*-*trans* isomer, 66%): δ 7.67–7.62 (*m*, 2H, PhH), 7.43 (*dd*, *J* = 8.1, 1.4 Hz, 1H, PhH), 7.21–7.01 (*m*, 4H, PhH), 6.90–6.84 (*m*, 1H, PhH), 4.11 (*d*, *J* = 8.3 Hz, 1H, CH_2_), 3.93–3.90 (*m*, 3H, CH_2_), 2.90 (*s*, 3H, C≡CCH_3_), 2.46 (*s*, 3H, C≡CCH_3_), 1.63 (*s*, 3H, CH_3_), 1.34 (*s*, 3H, CH_3_), 0.77 (*s*, 3H, CH_3_), 0.58 (*s*, 3H, CH_3_). IR (cm^−1^): 2995 (*w*), 2962 (*w*), 2928 (*w*), 2916 (*w*), 2894 (*w*), 1898 (*s*, C≡O), 1856 (*m*, C≡O), 1590 (*s*), 1572 (*s*), 1539 (*m*, C=N), 1455 (*m*), 1357 (*m*), 1326 (*m*), 1280 (*m*), 1246 (*m*), 1208 (*m*), 1160 (*m*), 1139 (*m*), 1053 (*s*), 966 (*m*), 818 (*m*), 776 (*m*), 741 (*s*), 695 (*m*), 653 (*m*). EI–MS (70 eV) *m*/*z*: [*M* – 2CO + O]^+^ 526.1.

### Refinement

Crystal data, data collection, and structure refinement details are summarized in Table 1 ▸. The H atoms of the CH_2_ groups were placed at positions with approximately tetra­hedral angles and C—H distances of 0.99 Å, and common isotropic displacement parameters were refined for the H atoms of the same group. The H atoms of the arene rings were placed at the external bisectors of the C—C—C angles at C—H distances of 0.95 Å, and common isotropic displacement parameters were refined for the H atoms of the same ring. The H atoms of the methyl groups were refined with common isotropic displacement parameters for the H atoms of the same group and idealized geometries with tetra­hedral angles, enabling rotations around the C—C bonds, and with C—H distances of 0.98 Å.

## Results and discussion

### Crystal structure analysis

**Figure 1:**
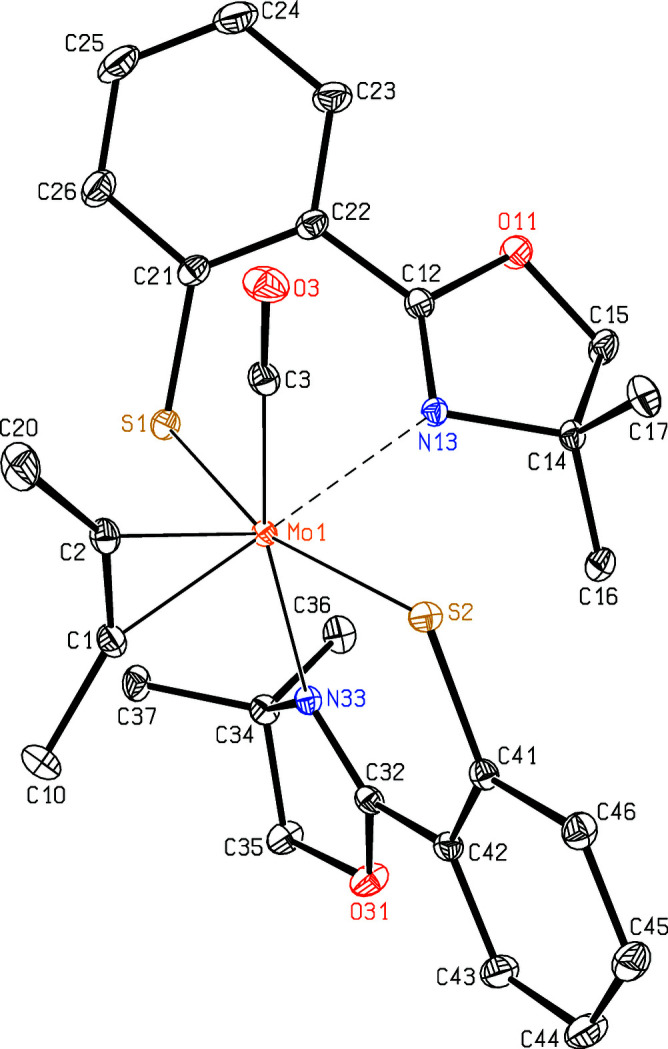
The mol­ecular structure of isomer **1**. Displacement ellipsoids are drawn at the 50% probability level and H atoms have been omitted for clarity. The rather long Mo—N distance [Mo1—N13 = 2.4715 (10) Å] is indicated by a dashed line.

**Figure 2:**
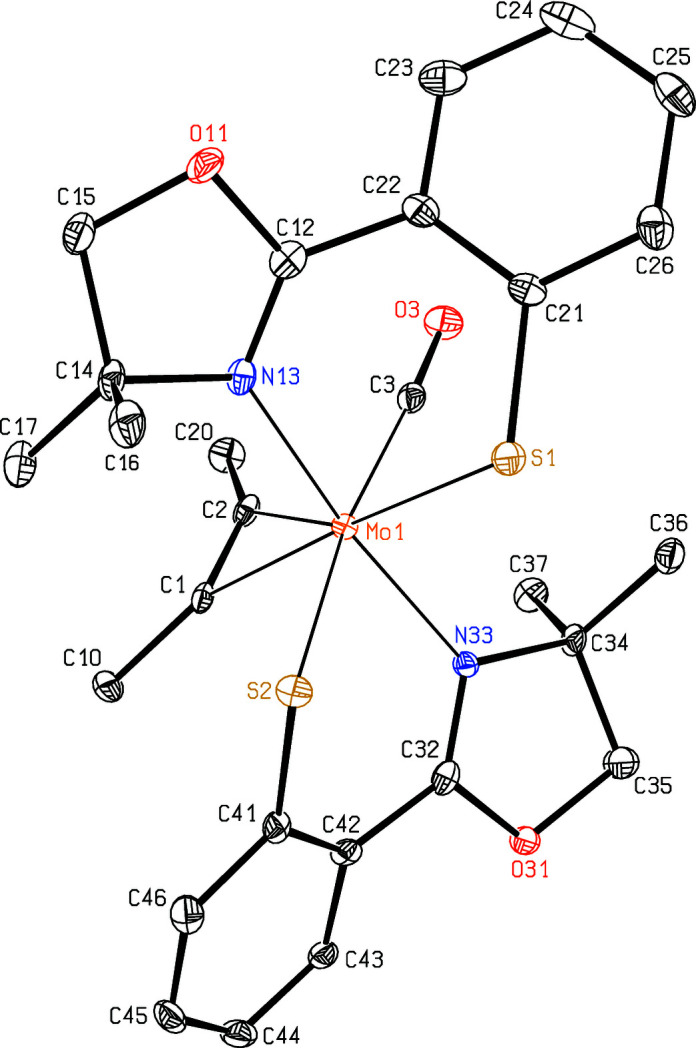
The mol­ecular structure of isomer **2**. Displacement ellipsoids are drawn at the 50% probability level and H atoms have been omitted for clarity.

Isomers **1** and **2** crystallize without any solvent mol­ecules in the monoclinic space groups *P*2_1_/*n* and *P*2_1_/*c*, respectively, and both have one metal com­plex in the asymmetric unit. In *N*,*N*-*cis* isomer **1** (Fig. 1 ▸), the Mo—N distance of the oxazole ring *trans* to the butyne ligand [Mo1—N13 = 2.4715 (10) Å] is much longer than that *trans* to the carbonyl ligand [Mo1—N33 = 2.3404 (11) Å]. In *N*,*N*-*trans* isomer **2** (Fig. 2 ▸), these distances [Mo1—N13 = 2.236 (2) Å and Mo1—N33 = 2.203 (2) Å] are com­parable to those observed in the dicarbonyl derivative [2.2333 (9) Å; Peschel *et al.*, 2013 ▸] or in the isotypic W com­pound [W1—N13 = 2.2153 (16) Å and W1—N33 = 2.1862 (16) Å; Peschel *et al.*, 2019 ▸]. In contrast to this, the Mo—S distances of the benzene­thiol­ate residues in isomer **1** are significantly different, although they are *trans* to one another, and both are clearly shorter [Mo1—S1 = 2.4673 (3) Å and Mo1—S2 = 2.3665 (3) Å] than in isomer **2** [Mo1—S1 = 2.5254 (8) Å and Mo1—S2 = 2.5297 (8) Å] or in the W com­pound [W—S = 2.5232 (4)–2.5243 (4) Å]. On the other hand, in both isomers, the distances are almost the same between the central atom and the butyne ligands [2.0310 (12)–2.0664 (12) *versus* 2.024 (3)–2.059 (3) Å] and to the carbonyl ligands [1.9417 (13) *versus* 1.953 (3) Å], although both are arranged in *trans* positions with respect to the N atoms of the oxazole rings in **1**, and *trans* to the S atoms of the benzene­thiol­ate groups in **2**. In both isomers, the CO ligands [C3—O3 = 1.1555 (16) and 1.157 (3) Å] lie roughly in the best planes through the butyne ligands [C1—C2 = 1.2965 (18) and 1.314 (4) Å] and the Mo atoms.

Comparing all known structures of *M*(CO)(C_2_ *R*_2_)(S-Phoz)_2_ com­plexes (Table 2 ▸), the following conclusions can be made: whereas *N*,*N*-*trans* conformations for *R* = H and CH_3_, and *S*,*S*-*trans* conformations for *R* = Ph were observed (Peschel *et al.*, 2015*a* ▸, 2019 ▸) for the W com­plexes, both conformations were found in the first two crystal structures of the analogous Mo com­plexes with *R* = CH_3_. In general, the Mo—N distances are clearly longer in the *S*,*S*-*trans* conformers, and slightly longer for the S-Phoz ligands *trans* to the alkyne ligands than those *trans* to the carbonyl ligand (*e.g. M*—N13 is larger than *M*—N33). In isomer **1**, the Mo—N distance of the S-Phoz ligand *trans* to the butyne ligand is exceptionally large due to the wide C1—Mo1—N13 angle of 173.53 (4)° and large C—*M*—S1 angles. The Mo—S distances are the same in the *N*,*N*-*trans* conformers, but in the *S*,*S*-*trans* conformers, *M*—S1 is distinctly longer than *M*—S2. Therefore, the S-Phoz ligands whose oxazole rings are *trans* to the alkyne ligands are more weakly bound to the metal centre than the others. In all six com­plexes (Table 2 ▸), the *M*—C1 distance is significantly shorter than *M*—C2, presumably due to the carbonyl ligand near atom C2.

### NMR spectroscopy

^1^H NMR spectra recorded in CD_2_Cl_2_ and CD_3_CN show a 1:2 ratio of the two isomers of Mo(CO)(C_2_Me_2_)(S-Phoz)_2_, while a 1:1 ratio is observed in CDCl_3_. The NMR data of isomer **2**, which presumably adopts the *N*,*N*-*trans* configuration, are almost identical with those of the W analogue (Peschel *et al.*, 2019 ▸), of which only the *N*,*N*-*trans* isomer was crystallized. In CD_2_Cl_2_ solutions, the two isomers of W(CO)(C_2_Me_2_)(S-Phoz)_2_ exhibit a 95:5 ratio, with a clear preference for the *N*,*N*-*trans* configuration of isomer **2**.

### IR spectroscopy

The IR spectrum of an average sample of Mo(CO)(C_2_Me_2_)(S-Phoz)_2_ shows a very strong band at 1898 cm^−1^ which is attributed to the C≡O bond. Due to weaker π-backbonding of the Mo centre, this bond is stronger by 18 cm^−1^ com­pared to that in the respective W com­pound (Peschel *et al.*, 2019 ▸), which is in accordance with previous observations on Mo and W carbonyl com­plexes (Ehweiner *et al.*, 2021*a* ▸,*b* ▸,*c* ▸). Despite the existence of two isomers, only one C≡O bond is visible.

## Supplementary Material

Crystal structure: contains datablock(s) 1, 2, global. DOI: 10.1107/S2053229622002029/wv3008sup1.cif

Structure factors: contains datablock(s) 1. DOI: 10.1107/S2053229622002029/wv30081sup2.hkl

Structure factors: contains datablock(s) 2. DOI: 10.1107/S2053229622002029/wv30082sup3.hkl

CCDC references: 2153636, 2153635

## Figures and Tables

**Table 1:** Experimental details For both structures: [Mo(C_11_H_12_NOS)_2_(C_4_H_6_)(CO)], *M*_r_ = 590.59, *Z* = 4. Experiments were carried out at 100 K with Mo *K*α radiation using a Bruker APEXII CCD diffractometer. Absorption was corrected for by multi-scan methods (*SADABS*; Bruker, 2013 ▸). Refinement was on 332 parameters. Only H-atom displacement parameters were refined.

	(1)	(2)
Crystal data		
Crystal system, space group	Monoclinic, *P*2_1_/*n*	Monoclinic, *P*2_1_/*c*
*a*, *b*, *c* (Å)	10.6159 (5), 8.9300 (4), 27.3801 (12)	9.1512 (4), 21.3515 (12), 13.1781 (7)
β (°)	96.189 (2)	98.483 (3)
*V* (Å^3^)	2580.5 (2)	2546.7 (2)
μ (mm^−1^)	0.70	0.71
Crystal size (mm)	0.18 × 0.18 × 0.10	0.23 × 0.07 × 0.07

Data collection		
*T*_min_, *T*_max_	0.884, 1.000	0.776, 1.000
No. of measured, independent and observed [*I* > 2σ(*I*)] reflections	30042, 11363, 9549	22009, 7415, 5339
*R*_int_	0.029	0.068
(sin θ/λ)_max_ (Å^−1^)	0.807	0.703

Refinement		
*R*[*F* ^2^ > 2σ(*F* ^2^)], *wR*(*F* ^2^), *S*	0.028, 0.071, 1.04	0.043, 0.087, 1.01
No. of reflections	11363	7415
Δρ_max_, Δρ_min_ (e Å^−3^)	0.72, −0.64	0.52, −0.83

Computer programs: *APEX2* (Bruker, 2013 ▸), *SAINT* (Bruker, 2013 ▸), *SHELX97* (Sheldrick, 2008 ▸), *SHELXL2014* (Sheldrick, 2015 ▸) and modified *ORTEP* (Johnson, 1965 ▸).

**Table 2:** Selected geometric parameters (Å, °) for *M*(CO)(C_2_ *R*_2_)(S-Phoz)_2_ com­plexes The labels C1 and C2 of the alkyne ligand were choosen such that the torsion angle C2—C1—*M*—C3 is approximately 0°. The selected ligand containing atoms S1 and N13 was that in which one of these atoms is *trans* to the alkyne ligand.

*M*, *R*	W, H^*a*^	W, CH_3_^*b*^	Mo, CH_3_^*c*^	Mo, CH_3_^*c*^	W, Ph^*b*^	W, Ph^*b*^
	*N*,*N*-*trans*	*N*,*N*-*trans*	*N*,*N*-*trans*	*S*,*S*-*trans*	*S*,*S*-*trans*	*S*,*S*-*trans*
*M*—C1	2.0268 (17)	2.0210 (17)	2.024 (3)	2.0310 (12)	2.0510 (19)	2.036 (4)
*M*—C2	2.0548 (18)	2.0565 (17)	2.059 (3)	2.0664 (12)	2.078 (2)	2.057 (4)
*M*—C3	1.9623 (18)	1.9535 (19)	1.953 (3)	1.9417 (13)	1.949 (2)	1.966 (4)
C3—O3	1.160 (2)	1.164 (2)	1.157 (3)	1.1555 (16)	1.155 (3)	1.154 (5)
*M*—N13	2.2120 (14)	2.2153 (16)	2.236 (2)	2.4715 (10)	2.3087 (18)	2.350 (3)
*M*—N33	2.1987 (14)	2.1862 (16)	2.203 (2)	2.3404 (11)	2.2975 (17)	2.304 (4)
*M*—S1	2.5050 (4)	2.5232 (4)	2.5254 (8)	2.4673 (3)	2.4620 (5)	2.4741 (12)
*M*—S2	2.5067 (4)	2.5243 (4)	2.5297 (8)	2.3665 (3)	2.3698 (5)	2.3773 (11)
C1—C2	1.327 (3)	1.314 (3)	1.314 (4)	1.2965 (18)	1.309 (3)	1.305 (6)
						
N13—*M*—N33	169.58 (5)	167.56 (6)	168.04 (8)	92.41 (3)	83.29 (6)	86.47 (13)
S1—*M*—S2	78.869 (14)	78.972 (15)	79.54 (3)	162.979 (11)	175.564 (18)	169.56 (4)
C1—*M*—N13	92.88 (6)	97.14 (7)	96.97 (9)	173.53 (4)	165.94 (7)	169.64 (15)
C2—*M*—N13	93.66 (7)	94.92 (6)	94.67 (9)	146.80 (4)	150.09 (7)	148.68 (15)
C3—*M*—N33	94.24 (6)	94.52 (7)	94.51 (9)	168.19 (4)	159.92 (8)	164.04 (15)
C1—*M*—S1	164.79 (6)	164.06 (5)	163.76 (8)	97.54 (3)	85.61 (5)	91.62 (13)
C2—*M*—S1	153.79 (6)	156.79 (5)	156.87 (8)	96.29 (3)	87.98 (5)	89.33 (12)
C3—*M*—S2	163.06 (5)	166.23 (5)	167.27 (9)	85.88 (4)	87.58 (6)	87.74 (14)

References: (*a*) Peschel *et al.* (2015*a* ▸); (*b*) Peschel *et al.* (2019 ▸); (*c*) this work.
